# Osimertinib Resistance via Histologic Transformation From Non-small Cell Lung Carcinoma to Carcinosarcoma

**DOI:** 10.7759/cureus.59293

**Published:** 2024-04-29

**Authors:** Paul Stegelmeier, James A Dawson, McKenzie Wallace, Magda Esebua

**Affiliations:** 1 Department of Pathology and Anatomical Sciences, A.T. Still University Kirksville College of Osteopathic Medicine, Kirksville, USA; 2 Department of Pathology and Anatomical Sciences, University of Missouri School of Medicine, Columbia, USA

**Keywords:** osimertinib resistance, tyrosine kinase inhibitor (tki), pulmonary carcinosarcoma (pcs), non-small cell lung carcinoma (nsclc), histologic transformation, osimertinib

## Abstract

Resistance to tyrosine kinase inhibitors (TKIs) in non-small cell lung carcinoma (NSCLC) remains a significant clinical challenge. Osimertinib, a third-generation TKI, has demonstrated efficacy in overcoming resistance, but novel resistance mechanisms continue to emerge. This case report presents a unique instance of histologic transformation from NSCLC to carcinosarcoma, representing a previously unreported manifestation of osimertinib resistance. We describe the clinical course of a 63-year-old female with epidermal growth factor receptor (EGFR)-mutant NSCLC who initially responded to osimertinib but eventually developed carcinosarcoma. The transformation was associated with additional EGFR mutations and alterations in RB and TP53. Despite aggressive treatment, the patient's condition deteriorated, emphasizing the limited therapeutic options for carcinosarcoma. This case underscores the need for further research to elucidate the molecular mechanisms behind histologic transformation and explore novel therapeutic strategies to address osimertinib resistance in NSCLC. Understanding and addressing these mechanisms are crucial for improving outcomes in patients facing this challenging form of resistance.

## Introduction

Despite the established efficacy of first- and second-generation TKIs in the treatment of NSCLC, resistance to these agents can develop [1]. The most common mechanism of resistance is a T790M mutation in the epidermal growth factor receptor (EGFR) exon 20. Osimertinib, an irreversible third-generation TKI, has proven efficacious despite this mutation. However, patients can develop acquired resistance to osimertinib within the first two years of use via heterogeneous and various possible mechanisms. One possible mechanism by which resistance to osimertinib develops is via histologic transformation [2]. Here we present a case of histologic transformation from non-small cell lung carcinoma (NSCLC) to carcinosarcoma, possibly representing a new manifestation of osimertinib resistance. The EGFR plays a crucial role in promoting cell survival and proliferation through the activation of downstream signaling pathways [3,4]. Mutations in the EGFR gene are commonly observed in various epithelial neoplasms, including lung adenocarcinoma. The most prevalent EGFR mutations involve in-frame deletions in exon 19 and a point mutation in exon 21 (L858R), together accounting for approximately 90% of EGFR alterations [5]. These discoveries have led to the development of EGFR tyrosine kinase inhibitors (TKIs) as potential therapies for lung cancer. Currently, five TKIs (erlotinib, gefitinib, afatinib, dacomitinib, and osimertinib) have received FDA approval for the treatment of EGFR-mutant NSCLC. Osimertinib has emerged as the first-line therapy, demonstrating the longest progression-free survival among third-generation EGFR TKIs [6].

Resistance to osimertinib can occur due to acquired resistance mechanisms, which can be categorized as EGFR-dependent or EGFR-independent [1,2]. These mechanisms include acquired EGFR mutations, amplifications, genetic fusions, MAPK-PI3K mutations, cell cycle gene alterations, and histologic transformation [2]. Histologic transformation involves the transformation of adenocarcinoma to another histological subtype of lung carcinoma; previous studies have observed transformations to small cell carcinoma or squamous cell carcinoma. 

Histologic transformation from NSCLC to small cell lung cancer (SCLC) arises in approximately 4-15% of cases and is associated with poorer patient prognosis. Overall survival with standard chemotherapy for SCLC is reported to be 14-20 months. SCLC transformation from adenocarcinoma has been associated with a comparatively worse survival of 7.1 months following TKI therapy. While the transformation of adenocarcinoma to small cells or squamous cell carcinoma is well-established, no cases of transformation to carcinosarcoma have been reported [7,8]. Given the relatively aggressive nature of carcinosarcomas in general, it is reasonable that transformation to this histopathology represents an important mechanism of osimertinib resistance. 

Increasing resistance to the otherwise effective and well-tolerated pharmacologic interventions for NSCLC poses a significant clinical challenge. With the emergence of resistance, the efficacy of TKIs against NSCLC diminishes progressively; hence, gaining a deeper comprehension of resistance mechanisms to the remaining effective TKIs, like osimertinib, becomes crucial. While histologic transformation has been previously reported, we present a novel histological transformation from adenocarcinoma to carcinosarcoma, leading to resistance to osimertinib and disease progression [2]. This case represents an important and detrimental mechanism of resistance. 

## Case presentation

A 63-year-old female with no history of smoking presented with fecal incontinence, debilitating headaches, right arm weakness, gait instability, and a recent ground-level fall. Imaging studies revealed an 8 cm area of cerebral edema in the left parietal lobe, a 3.5 cm mass in the superior segment of the lower lobe of the right lung, numerous subcentimeter nodules throughout the lung parenchyma, and hilar lymphadenopathy. A CT-guided lung core needle biopsy confirmed invasive NSCLC.

**Figure 1:**
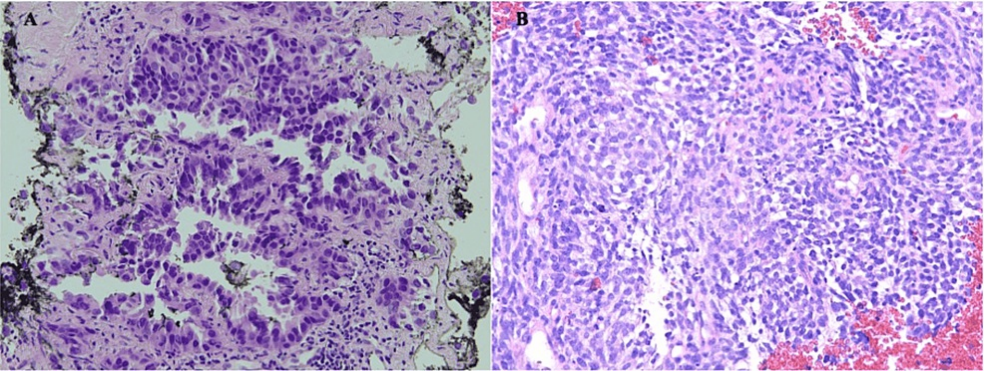
Comparison of lung mass biopsy at initial diagnosis and after treatment. A) Lung mass biopsy at initial diagnosis (H&E, 200x) demonstrating lung adenocarcinoma with a predominantly lepidic pattern. B) Lung mass biopsy after 17 months of treatment with osimertinib (H&E, 200x) demonstrating lung carcinosarcoma with islands of sarcomatoid spindled cells among solid epithelioid components.

**Figure 2:**
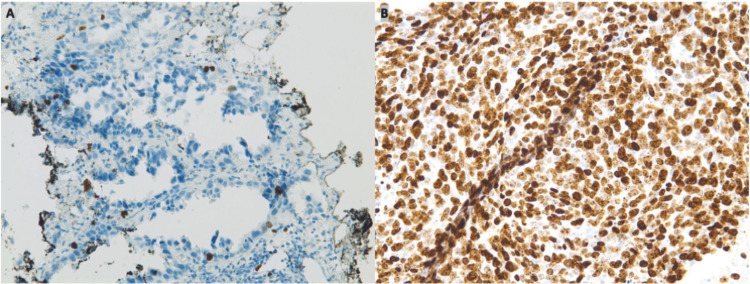
Comparison of Ki-67 staining in primary lung mass and lymph node. A) Biopsy of primary lung mass at initial diagnosis (Ki-67, 200x) showing positive staining in <5% of cells. B) Fine-needle aspiration of 11R lymph node after 17 months of treatment with osimertinib (Ki-67, 200x) showing Ki-67 positivity in >90% of cells.

The primary histology showed neoplastic cells extending along alveolar epithelia, producing a prominent lepidic pattern (Figure 1A). Immunohistochemical analysis demonstrated positive staining for TTF-1, Napsin A, CK7, and CK5/6, and negative staining for CK20, P40, chromogranin, and synaptophysin. The cell proliferation index was estimated to be less than 3%, via Ki-67 stains (Figure 2). Genpath OnkoSight histologic and tissue tumor next-generation sequencing revealed an EGFR Glu 757-Ala 750 deletion mutation, a rare exon 19 deletion variant.

Once the diagnosis was confirmed, the patient underwent stereotactic radiosurgery (SRS) and initiated treatment with osimertinib 80 mg daily. The patient demonstrated treatment response initially; however, after more than 17 months of treatment, follow-up imaging showed progressive and recurrent brain and chest lesions. Endobronchial ultrasound-guided fine-needle aspiration of the 11R lymph node confirmed a poorly differentiated malignancy consistent with metastatic carcinosarcoma.

Further characterization of the metastatic recurrent lung cancer revealed a biphasic morphology with solid sheets of pleomorphic, neoplastic mesenchymal cells interspersed with small islands of neoplastic epithelial cells (Figure 1B). Immunocytochemistry now demonstrated positive staining for TTF-1, AE1/AE3, EMA, CD56, and vimentin with negative staining for CK7, P40, napsin, calretinin, PAX-8, S-100, synaptophysin, and chromogranin. Ki-67 staining revealed an estimated cell proliferation index of 90% of the cells (Figure 2). Foundation one next-generation sequencing identified an additional EGFR exon 19 deletion (E746_A750del) in addition to the previously identified EGFR G796C and 1744V point mutations. Notable novel mutations were also found in RB (R552) and TP53 (C275F), with a tumor mutational burden greater than 10 Mut/Mb. The microsatellite status was characterized as equivocal.

Once transformation to carcinosarcoma was confirmed, osimertinib was discontinued and the patient received four cycles of carboplatin, pembrolizumab, and pemetrexed every three weeks. Pembrolizumab was held during the fourth cycle due to transaminitis. Unfortunately, the patient's clinical status continued to deteriorate, with the development of multiple new brain metastases, and is currently receiving palliative care.

## Discussion

This case report highlights the development of histologic transformation from NSCLC to carcinosarcoma as a novel and important mechanism of acquired resistance to osimertinib. Histologic transformation is a recognized phenomenon in various malignancies and has previously been associated with resistance to targeted therapies; it is, therefore, plausible that the current case of histologic transformation represents a development of resistance to osimertinib. The exact mechanisms underlying this histologic transformation remain poorly understood and warrant further investigation; however, it is believed to result from clonal evolution, genomic instability, and selective pressure from treatment. In this case, the acquisition of additional EGFR exon 19 deletion mutations, as well as alterations in RB and TP53, suggests genomic evolution and increased tumor heterogeneity likely contributed to the development of carcinosarcoma.

Osimertinib-associated histologic transformation poses a significant challenge due to the aggressive and associated poor prognosis of these tumors. In this case, due to the poor response to treatment, osimertinib was discontinued and followed by an aggressive chemotherapy regimen including carboplatin, pembrolizumab, and pemetrexed. Despite treatment modifications, the patient experienced disease progression, highlighting the limited therapeutic options for carcinosarcoma.

In considering the evolution of this case, it is important to acknowledge the possibility of coexisting carcinosarcoma in addition to adenocarcinoma that may have not been sampled on initial diagnosis. The histologic transformation observed here may represent a manifestation of this dual pathology, contributing to the resistance observed with osimertinib treatment. The coexistence of multiple tumor subtypes within the same lesion is not uncommon in lung cancer and can present challenges in accurate diagnosis and treatment planning. Therefore, it is plausible that the initial biopsy may have captured only a portion of the tumor heterogeneity, underestimating the complexity of the disease. Future studies employing more comprehensive tissue sampling techniques, such as multi-region biopsies, may shed light on the true extent of tumor heterogeneity and aid in the early detection of histologic transformations.

Further research is needed to better understand the molecular mechanisms underlying histologic transformation and identify potential therapeutic strategies to overcome resistance in such cases. Additionally, studies exploring the utility of comprehensive genomic profiling to identify early signs of transformation, guide treatment decisions, and potentially prevent further development of resistance are warranted.

## Conclusions

Histologic transformation to carcinosarcoma represents a possible manifestation of acquired resistance to osimertinib in NSCLC. This case report illustrates the clinical challenges resulting from osimertinib resistance mechanisms that lead to phenotypic changes and limit treatment options. Future research should focus on elucidating the mechanisms driving histologic transformation and identifying novel therapeutic approaches to improve outcomes for patients experiencing this form of resistance.
